# Conducted dilatation to ATP and K ^+^ in rat skeletal muscle arterioles

**DOI:** 10.1111/apha.12656

**Published:** 2016-02-22

**Authors:** K. A. Dora

**Affiliations:** ^1^ Department of PharmacologyUniversity of OxfordOxfordUK

**Keywords:** conducted dilatation, cremaster, electrical coupling, K_ATP_ channel, K_Ca_ channel, K_IR_ channel

## Abstract

**Aim:**

During exercise in humans, circulating levels of ATP and K^+^ increase at a time when blood flow increases to satisfy metabolic demand. Both molecules can activate arteriolar K^+^ channels to stimulate vasodilatation; here, it is established whether conducted dilatation is observed in a skeletal muscle bed.

**Methods:**

Isolated and cannulated rat cremaster arterioles were used to assess both local and conducted responses. Agents were either added to the bath, focally pulse‐ejected to the downstream end of arterioles, or in triple‐cannulated arterioles, luminally perfused into the downstream branches to assess both local and conducted responses.

**Results:**

The endothelium‐dependent agonist ACh and the K_ATP_ channel opener levcromakalim each stimulated both local and conducted vasodilatation. Focal, bolus delivery of ATP (10 *μ* m) or KCl (33 mm) to the outside of arterioles stimulated a biphasic vasomotor response: rapid vasoconstriction followed by dilatation as each washed away. At lower concentrations of KCl (19 mm), constriction was avoided, and instead, Ba^2+^‐sensitive local dilatation and conducted dilatation were both observed. Luminal perfusion of ATP avoided constriction and activated P2Y_1_ receptors stimulating vasodilatation secondary to opening of K_C__a_ channels. In triple‐cannulated arterioles, either ATP (10 *μ* m) or K^+^ (15 mm) luminally perfused into daughter branches of a bifurcation stimulated local dilatation which conducted into the parent arteriole.

**Conclusion:**

The recognized physiological autocrine and paracrine mediators ATP and K^+^ each act to evoke both local and conducted vasodilatation in rat cremaster arterioles. Therefore, in situations when circulating levels are raised, such as during exercise, these agents can act as important regulators of blood flow.

The control of skeletal muscle blood flow is regulated by the extent of tone generated by intraluminal pressure (causing myogenic tone) and vasoconstrictors, balanced by the release of vasodilators from cells both within the artery wall and surrounding tissue (Clifford & Hellsten 2004, Clifford 2007). The use of the cremaster preparation for intravital studies *in vivo* clearly demonstrates that either reducing the PO_2_ across the tissue or electrical stimulation of skeletal muscle bundles can each stimulate vasodilatation of arterioles (Klitzman *et al*. 1982, Hester & Duling 1988, Segal & Duling 1989, Cohen *et al*. 2000, Cohen & Sarelius 2002). The vasodilator mediators responsible are not fully elucidated, but candidates include (i) ACh released from motor nerve endplates (Welsh & Segal 1997); (ii) ATP released from hypoxic erythrocytes (Ellsworth *et al*. 2009); (iii) K^+^ released through open K^+^ channels, for example during repolarization of skeletal muscle fibres (Armstrong *et al*. 2007, Crecelius *et al*. 2014); and (iv) activation of K_ATP_ channels by low intracellular (ATP) (Cohen & Sarelius 2002, Ngo *et al*. 2010). How the dilators influence blood flow upstream beyond the area they directly stimulate is thought to require conducted vasodilatation, which significantly reduces vascular resistance to affect increases in blood flow (Segal & Jacobs 2001, Segal 2005). This phenomenon has been shown to occur in response to a variety of vasodilators, the most commonly studied being the endothelium‐dependent vasodilator, ACh, which hyperpolarizes smooth muscle cells secondary to opening endothelial Ca^2+^‐activated K^+^ (K_Ca_) channels as well as releasing nitric oxide. Having no action at smooth muscle cells, ACh can be delivered from pulled micropipettes by iontophoresis or pressure‐pulse ejection abluminally onto defined, localized areas of the preparation, whereupon the stimulated dilatation conducts upstream through the arteriolar wall due to electrical coupling (Welsh & Segal 1997, Emerson & Segal 2000a, Hoepfl *et al*. 2002, Wölfle *et al*. 2011). The conducted dilatation facilitates a drop in resistance of sufficient magnitude to increase blood flow through small arteries and arterioles to match flow and metabolic demand (Duling & Berne 1970, Williams & Segal 1993, Kurjiaka & Segal 1995, Dora *et al*. 2000).

Conducted dilatation depends on a hyperpolarizing stimulus. Following hyperpolarization in response to agonist activation of either endothelial cell K_Ca_ channels or smooth muscle cell ATP‐sensitive K^+^ (K_ATP_) channels (Delashaw & Duling 1991, Emerson & Segal 2000a, Takano *et al*. 2004), hyperpolarization spreads longitudinally between coupled endothelial cells, and then to the surrounding smooth muscle cells along the artery length (reviewed in Domeier & Segal 2007). Most studies highlight the importance of the endothelium as the conduit for increases in membrane potential (Haas & Duling 1997, Yamamoto *et al*. 1999, Emerson & Segal 2000b, Takano *et al*. 2004, Winter & Dora 2007) and the importance of the gap junction protein connexin 40 (de Wit *et al*. 2000, Figueroa & Duling 2008). Generally, the conducted dilatation is not altered in the presence of a nitric oxide synthase (NOS) inhibitor (Domeier & Segal 2007, Winter & Dora 2007).

However, despite being important physiological vasodilators, studying conducted vasodilatation to ATP and KCl is more complicated because each can also stimulate contraction of smooth muscle when applied to the outside of arterioles, especially at the higher concentrations usually present in stimulating pipettes. Therefore, the aim of this study was to demonstrate whether moderate and physiologically relevant increases in extracellular K^+^ concentration and ATP stimulate conducted dilatation responses in rat cremaster arterioles. This was achieved by longer duration syringe‐pump delivery of low concentrations of each agonist to the downstream end of arterioles, either to the outside of arterioles via pipettes or directly into the lumen of cannulated daughter branches at a bifurcation. Overall, these data demonstrate that activation of K_Ca_ channels by ATP and K_IR_ channels by KCl can each stimulate robust local dilatation and initiate conducted dilatation in skeletal muscle, mechanisms which *in vivo* will improve blood flow to match increases in metabolic demand.

## Materials and methods

### Tissue preparation

All animal usage conformed with the Animals (Scientific Procedures) Act 1986 and was approved by the University of Oxford Local Ethical Review Committee and the UK Home Office. Male Wistar rats (Charles River, UK; 240–280 g) were anaesthetized with urethane (2.8 g kg^−1^ i.p.). The cremaster muscle was excised and placed in ice‐cold Mops‐buffered solution containing (in mm): NaCl 145.0, KCl 4.7, CaCl_2_ 2.0, MgSO_4_·7H_2_O 1.17, Mops 2.0, NaH_2_PO_4_ 1.2, glucose 5.0, pyruvate 2.0, EDTA 0.02, NaOH 2.75 (pH was adjusted to 7.40 ± 0.02 at 37 °C). Following tissue extraction, rats were killed by Schedule 1 protocols.

### Pressure myography

Arteriole segments (1A; Hill *et al*. 1992) were dissected from the cremaster muscle and cannulated with glass pipettes connected to a closed pressure system, as described previously (Winter & Dora 2007, Bagher *et al*. 2012). Each cannulating pipette was independently positioned using 3‐axis micromanipulators (U‐31CF; Narishige YOU Series, Tokyo, Japan) fixed to the *x*−*y* stage of an inverted microscope, which was connected to a confocal scanning unit (Olympus FV500, FV1000 or FV1200, Tokyo, Japan). The isolated arteriole was placed in a temperature‐controlled chamber (RC‐27 chamber, PH‐6 platform; Warner Instruments, Hamden, CT, USA) also fixed to the microscope stage, and temperature continuously monitored using a microprobe (IT‐1E; Physitemp Instruments, Clifton, NJ, USA). This enabled careful positioning of the arteriole, and increasing the length in each axis as the arteriole was pressurized. After heating to 34.5 °C, intraluminal pressure was increased to 80 mmHg and the arteriole was tested for leaks and allowed to equilibrate for 20 min. Only arterioles without leaks that developed 35–50% myogenic tone, fully dilated to the endothelium‐dependent agonist ACh (0.3–1 *μ* m) and recovered myogenic tone following ACh washout were used for experiments. A 10× objective (Olympus) was used for all experiments where diameter was measured and images were acquired at 1 Hz. For all conducted vasomotor experiments, simultaneous transmitted and fluorescence images were acquired to detect the direction of flow of agonist solutions, which always contained carboxyfluorescein (excitation 488 nm, emitted light >505 nm). In this way, the delivery of agonist was watched live, and any upstream spread of agonist was avoided. The lowest possible laser intensity was always used, in some cases aided by the use of high‐quantum‐efficiency peltier‐cooled gallium arsenide phosphide photomultiplier tube (Olympus FV1200). The preparations remained viable for many hours, assessed by maintenance of myogenic tone and dilatation to agonists.

### Local pressure‐pulse ejection of agonists

Agonists (ACh, LVK or ATP, each 10 *μ* m) in a Mops‐buffered solution containing carboxyfluorescein (250 nm) were backfilled into plain, pre‐pulled glass micropipettes with bevelled 5‐*μ*m tips (World Precision Instruments, Sarasota, FL, USA). The micropipette was positioned near the midplane of myogenically active arterioles, almost touching the outer wall. Soluti‐ons were pressure‐pulse‐ejected using a pneumatic pico‐pump (PV 820; World Precision Instruments at 5–10 psi for durations up to 500 ms). The pressure‐pulse delivery pipette was often moved away from the arteriole as soon as the pulse was ejected to avoid damaging the arteriole with the tip of the pipette as the arteriole dilated (Movie S1). Short‐duration pulses (100–500 ms) evoked near maximal responses.

To more reliably and reproducibly deliver agonists for longer periods of time in order to establish more stable responses, in separate experiments, pulled pipettes with larger inner diameter tips (approx. 30–50 *μ*m) were used to continuously pump (BeeHive^®^ syringe pump; Bioanalytical Systems, West Lafayette, IN, USA) ATP (10 *μ* m at 8 *μ*L min^−1^ for up to 10 s), isotonic KCl (33 mm varied between 1 and 8 *μ*L min^−1^ or 19 mm at 8 *μ*L min^−1^ for up to 20 s) or ACh (1 *μ* m at 1 *μ*L min^−1^ for up to 10 s). In all cases, before recording responses to agonists, the direction of superfusion flow along the arteriolar length was determined, as the cannulating pipettes caused turbulent and often unpredictable superfusion flow. In cases where superfusion flow was in the opposite direction to that expected based on the superfusion inflow line (due to turbulent flow around the cannulating pipette), the pulse ejection or pump pipette was positioned at the other end of arterioles. Experiments were only performed using preparations where the applied agonist (and carboxyfluorescein) only flowed downstream to the superfusion flow direction and did not directly reach arteriolar positions upstream from the delivery pipette.

When recording responses, agonists were focally delivered to the outside of arterioles to study local dilatation and conducted dilatation. Using a 10× objective, 1.2 mm lengths of arterioles were imaged in a field of view for simultaneous diameter and fluorescence measurements at positions 0–1000 *μ*m from the stimulating pipette.

### Luminal perfusion of agonists

In all experiments where agents were perfused into the lumen of arterioles, the cannulating pipette inner diameters were maximized (approx. 80 *μ*m) and the downstream pipette inner diameter was larger than that of the upstream pipette, each to avoid the generation of resistance within the arteriolar lumen. In all experiments, the luminal flow rate was set to below that causing damage to the arterioles, the latter assessed as irreversible flow‐induced vasodilatation. Using these flow rates, shear stress ranged from 0.5 to 50 dyn cm^−2^, and flow‐induced dilatation was not observed in any experiments. All pumped luminal solutions were driven by individual BeeHive^®^ syringe pumps, a custom‐built manifold near the cannulating pipette allowing multiple solutions to be readily used.

In experiments characterizing the responses to purines, data were normalized to the frame immediately before first observed change in diameter, designated *t *=* *0 s. Responses to multiple concentrations of ATP were obtained in each arteriole by pumping a Mops‐filled perfusion line between each concentration, allowing myogenic tone to reestablish between responses and generating non‐cumulative concentration–response curves. In some experiments, antagonists were also included in the luminal perfusion solutions.

To examine the contribution of Ca^2+^‐activated K^+^ channels to ATP‐stimulated vasodilatation, arterioles were pre‐incubated in 1‐[(2‐chlorophenyl)diphenylmethyl]‐1*H*‐pyrazole (TRAM‐34, 1 *μ* m, to block IK_Ca_), apamin (50 nm, to block SK_Ca_) and iberiotoxin (100 nm, to block BK_Ca_) cumulatively and 10 *μ* m ATP‐stimulated dilatation assessed after each addition. To identify the receptor primarily mediating the response to ATP, arterioles were pre‐incubated with the selective P2Y_1_ receptor antagonist, 2′‐deoxy‐*N* ^*6*^‐methyladenosine 3′,5′‐diphosphate diammonium salt (MRS 2179, 1 or 3 *μ* m, bath and luminal solutions). The selective, non‐hydrolyzable analogue of ADP, adenosine‐5′‐0‐(2‐thiodiphosphate) trilithium salt (ADP*β*S) was used as a P2Y_1_ agonist.

### Triple‐cannulated arterioles

Cremaster arterioles (1A, parent) with a bifurcation intact (1A and 2A, daughter branches 1 and 2) were isolated and triple‐cannulated (Hill *et al*. 1992, Winter & Dora 2007). When a small side branch was found just downstream of the bifurcation, it was avoided and the longer arteriole was then used to infuse agonists. Following preparation as above, arterioles with myogenic tone in the parent and daughter branch arterioles were used. Approximately 30–60 s before commencing perfusion of agonists into branch 1, intraluminal flow (approx. 5–10 *μ*L min^−1^) was introduced into the upstream end of the parent artery by generating a pressure gradient between the upstream and downstream pipettes as described previously (Winter & Dora 2007). This pressure gradient maintained an average intraluminal pressure of 80 mmHg, and the flow in itself did not alter arterial diameter. Agonist solutions contained carboxyfluorescein (250 nm) to monitor delivery and were pumped intraluminally into branch 1 of the bifurcation using a BeeHive^®^ syringe pump (1–8 *μ*L min^−1^). Continuous flow in the parent (feed) arteriole ensured cells upstream from the bifurcation were not exposed to the perfused drug.

Diameter values are expressed as raw inner diameter (*μ*m) or percentage of the maximum passive diameter. In some experiments (flow response and KCl conducted response), the BeeHive^®^ syringe pumps were used to deliver constant flow into both the parent and branch 1 arterioles, the pressure head held by the gravity‐fed perfusion line connected to branch 2. The flow rates used were low enough not to impact the pressure head to any significant degree: 0.5 and 1.0 *μ*L min^−1^ in the parent and daughter arterioles respectively. The surface area of the pressure reservoir syringe was approx. 95 mm^2^; therefore, it would take >1 h to raise this level by 1 mmH_2_O, <0.1 mmHg.

### Diameter measurements

For all measurements of diameter, arteriolar inner diameter was measured offline using motion analysis software (metamorph v7.7.4; Molecular Devices, Sunnyvale, CA, USA) or with manual distance analysis (imaris v7.7.1; Bitplane Scientific Software, Zurich, Switzerland). This enabled simultaneous analysis of multiple, calibrated distances along the artery wall, and direct comparisons of local dilatation to conducted dilatation for a single application of agonist, which is not possible with higher magnification objectives. The resolution of the system was 1.8 *μ*m (equivalent to one pixel), 3.6% of the smallest diameter measured. Myogenic tone (*D*_MT_) was calculated as the per cent decrease in diameter (*D*) from the maximum diameter (*D*_Max_) of each arteriole [(*D*_Max_−*D*)/*D*_Max_ × 100]. The dilatation evoked by each agonist (*D*_Agonist_) was calculated as the per cent of the maximum diameter from myogenic tone [(*D*_Agonist_−*D*)/(*D*_Max_−*D*) × 100]; vasoconstriction resulted in a negative value. Fluorescence intensity (*F*) was also measured offline simultaneously at multiple positions in the lumen of arteries, which was temporally matched to diameter measurements. Data were normalized to the maximum fluorescence intensity released from the delivery micropipette (*F*/*F*_Max_). Concentration–response curves, graphs and histograms were prepared using prism v5.0 software (GraphPad Software, La Jolla, CA, USA).

### Drugs and solutions

All drugs were obtained from Sigma (Poole, UK) with the exception of levcromakalim (R&D Systems, Abingdon, UK) and apamin (Latoxan, Valence, France). Raised KCl solutions were kept isotonic by equal substitution with NaCl. All stock solutions were prepared in purified water except levcromakalim, glibenclamide and TRAM‐34 (each 10^−2^ m in DMSO, TRAM‐34 further diluted to 10^−3^ m in DMSO)and then diluted in Mops‐buffered solution for use. The stock solutions of ATP (10^−2^ m) and 5(6)‐carboxyfluorescein (5 × 10^−4^ m) and the working solution of L‐NAME (*N* ^G^‐nitro‐l‐arginine methyl ester, 100 *μ* m) were adjusted to pH 7.4. Prior to use in experiments, all drugs were diluted in physiological buffer and kept chilled (approx. 4 °C); the vehicle had no effect. All inhibitors were incubated in the bath for a minimum of 20 min prior to obtaining responses, except for MRS 2179 (also added to the lumen of arterioles) and apamin (1 h).

### Statistical analysis

All data are summarized as the mean ± SEM from *n* arterioles, one per animal. Statistical comparisons were made using prism software and one‐way anova with Bonferroni post‐test where appropriate; *P *<* *0.05 was considered statistically significant.

## Results

The arterioles used in this study had a maximum diameter of 167 ± 16 *μ*m, *n *=* *41, measured at the mid‐point of parent arterioles (approx. 500 *μ*m upstream from the delivery pipette or bifurcation). When studied, the maximum diameter of (daughter) branch 1 arterioles used for agonist perfusion was 136 ± 27 *μ*m, *n *=* *13. The per cent myogenic tone in arterioles cannulated with two pipettes was 43.3 ± 5.5%, resulting in a baseline diameter of 94 ± 15 *μ*m (*n *=* *28), and in triple‐cannulated arterioles, the parent and branch 1 arterioles had 43.5 ± 5.0 and 40.4 ± 6.9% myogenic tone, with baseline diameters 97 ± 13 and 81 ± 21 *μ*m respectively (*n *=* *13).

### ACh and LVK stimulate conducted vasodilatation

**Figure 1:**
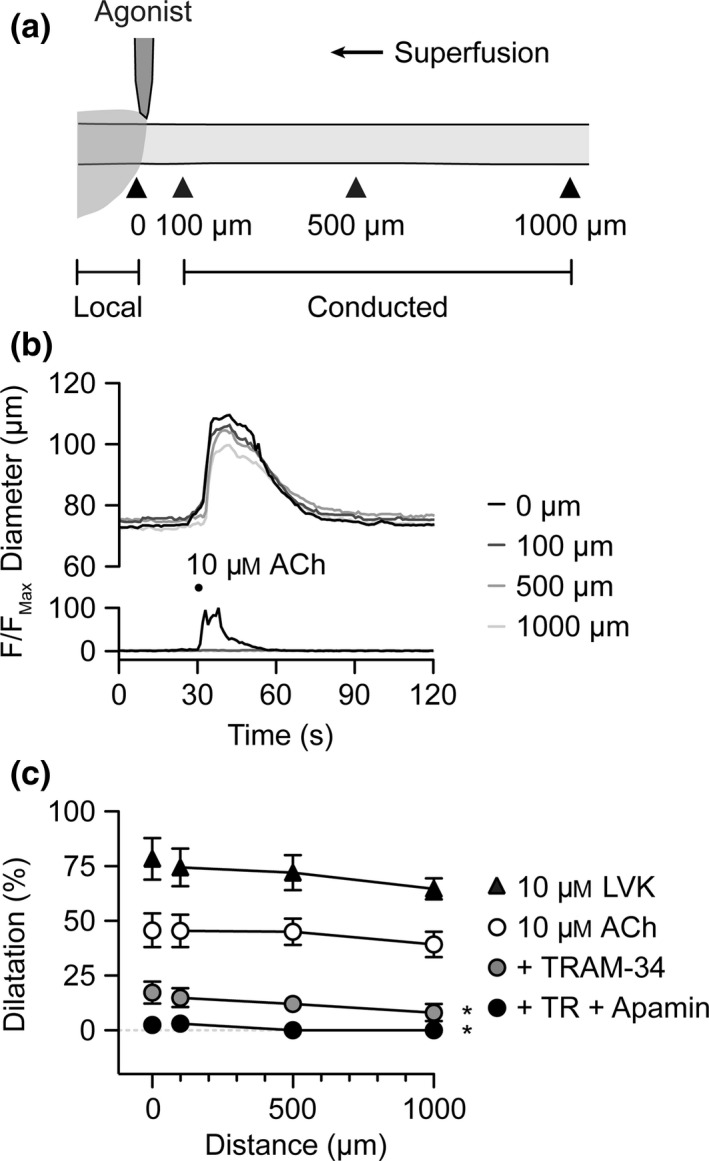
IK_C__a_ channels underlie conducted dilatation to pulsed ACh. (a) Schematic of experimental set‐up for abluminal pressure‐pulse ejection of agonists to cannulated arterioles. The delivery micropipette was positioned at the downstream end of the arteriole. Diameter was measured locally (0 *μ*m) and conducted responses at sites 100, 500 and 1000 *μ*m upstream (indicated by arrowheads). (b) Representative time course of changes in diameter and fluorescence intensity in response to pulsed solution containing ACh (10 *μ* m) and carboxyfluorescein. (c) Summary data showing local and conducted responses to ACh under control conditions (white circles, *n *=* *8), and during block of K_Ca_ channels by TRAM‐34 alone (*n *=* *3) and with apamin (*n *=* *3). In the same experiments, LVK (10 *μ* m) was subsequently pulsed to activate K_ATP_ channels (black triangles, *n *=* *3). *P <* *0.05 vs. 10 *μ* m ACh control. See Movie S1 for representative response to ACh.

When pressure‐pulse‐ejected to the outside of arterioles, both ACh and LVK stimulated local dilatation and conducted dilatation (Fig. 1, Movie S1). The response to ACh was inhibited locally and up to 1000 *μ*m upstream by TRAM‐34 (1 *μ* m, IK_Ca_ inhibitor), the residual dilatation blocked by apamin (50 nm, SK_Ca_ inhibitor). Vasodilatation to LVK is fully blocked with the K_ATP_ channel blocker glibenclamide (5 *μ* m) (Bagher *et al*. 2012).

### Abluminal stimulation with ATP stimulates a biphasic response

**Figure 2:**
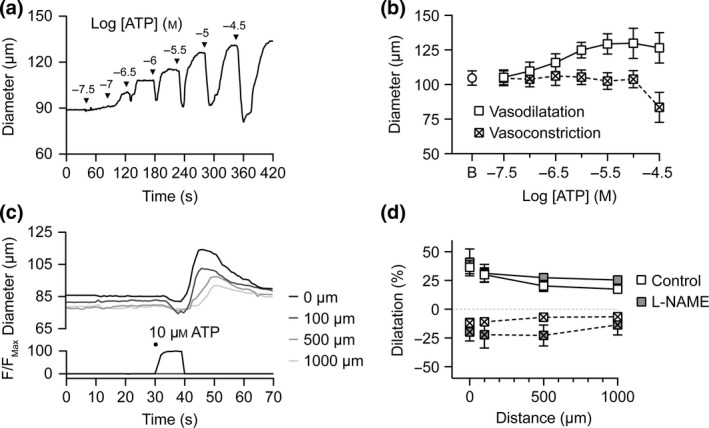
Biphasic vasomotor response to abluminal ATP. (a) Representative cumulative concentration–response curve to ATP added to the outside of an arteriole. Note the transient constriction, followed by dilatation. (b) Summary of diameter values at baseline (B) then peak constriction and peak dilatation responses to each concentration of ATP (*n *=* *7). (c) Representative time course of changes in diameter and fluorescence intensity in response to abluminally pumped solution containing ATP (10 *μ* m) and carboxyfluorescein. The micropipette was positioned at the downstream end of the arteriole and responses were measured at 0–1000 *μ*m upstream. (d) Summary data showing local and conducted constriction (squares with cross) and dilatation responses to ATP under control conditions (white squares, *n *=* *5); and lack of block by the nitric oxide synthase inhibitor L‐NAME (grey squares, *n *=* *3).

Cumulative concentration–response curves to bath‐applied ATP stimulated biphasic vasomotor responses (Fig. 2). At low concentrations (up to 100 nm), only dilatation was observed. At higher concentrations of ATP (0.3–30 *μ* m), rapid, transient contraction was followed by maintained dilatation. When applied focally for a few seconds using pressure‐pulse ejection, 10 *μ* m ATP stimulated a transient local and conducted contraction, followed by local dilatation and conducted dilatation. In these experiments, a wider‐tipped pipette was used to deliver ATP for a longer duration (up to 10 s) to improve the possibility of ATP reaching the endothelium. In all experiments, only a transient contraction to ATP was observed, and there was no clear difference in the magnitude of constriction or dilatation to ATP when compared to shorter, pressure‐pulse‐ejected ATP; therefore, these data were combined (Fig. 2d).

### Abluminal stimulation with KCl stimulates a biphasic response

**Figure 3:**
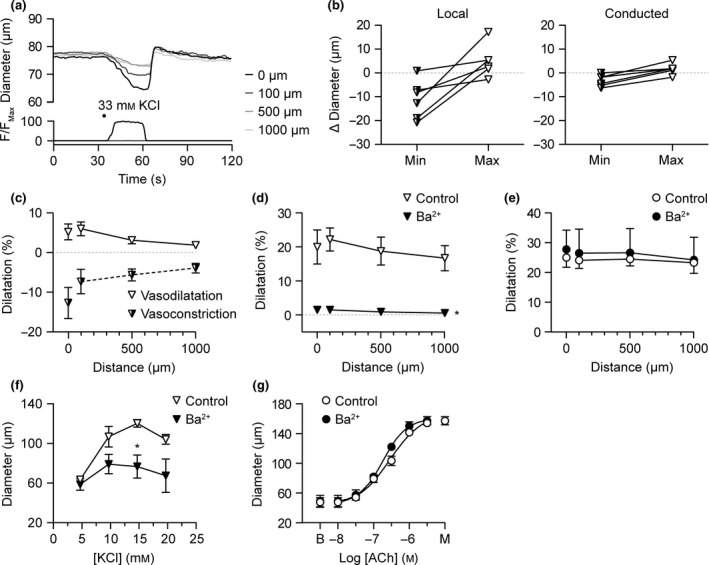
Biphasic vasomotor response to abluminal KCl. (a) Representative time course of changes in diameter and fluorescence intensity in response to abluminally pumped solution containing isotonic KCl (33 mm) and carboxyfluorescein. The micropipette was positioned at the downstream end of the arteriole and responses were measured at 0–1000 *μ*m upstream. (b) Summary data showing paired constriction (Min, half‐filled inverted triangles) and dilatation (Max, white inverted triangles) responses to 33 mm KCl under control conditions directly at the pipette (0 *μ*m, Local) and 1000 *μ*m upstream (Conducted; *n *=* *6 responses from three arterioles). (c) Summary data showing local and conducted constriction and dilatation responses to 33 mm KCl under control conditions (*n *=* *6, from three arterioles). (d) Summary data showing local and conducted dilatation responses to abluminally pumped solution containing isotonic 19 mm KCl under control conditions (white inverted triangles) and in the same arterioles, in the presence of 30 *μ* m Ba^2+^ (filled inverted triangles, *n *=* *3). No vasoconstriction was observed to this concentration of KCl. (e) Summary data showing local and conducted dilatation responses to abluminally pumped solution containing 1 *μ* m ACh under control conditions (white circles) and in the same arterioles, in the presence of 30 *μ* m Ba^2+^ (filled circles, *n *=* *3). In (d) and (e), the pipette positions were not changed before and after Ba^2+^. On completion of pipette‐applied responses, the responses to bath‐applied KCl (f) and ACh (g) were obtained in the same arterioles; 15 mm KCl stimulated 60.6 ± 1.0% of maximum diameter (*n *=* *3); B, baseline; M, maximum diameter. *P <* *0.05 vs. control.

We have previously shown that 15 mm KCl can stimulate approx. 60% dilatation of rat cremaster arterioles (McSherry *et al*. 2006), whereas higher concentrations stimulate constriction (Meininger & Faber 1991, Potocnik *et al*. 2000). The previous report demonstrating conducted dilatation to focal abluminally delivered (short‐duration pressure‐pulse‐ejected) KCl in cremaster (Hungerford *et al*. 2000) used 1 m KCl in the delivery pipette, and using this approach reported both constriction and dilatation to KCl. Here, using larger tip diameter pipettes and a longer duration delivery (by pumping), similar data were obtained using 33 mm KCl (isotonic solution), where local and conducted constriction were observed during the agonist delivery period, followed by local dilatation and conducted dilatation upon cessation of pumping (Fig. 3a). When the rate of pumping was varied between 1 and 8 *μ*L min^−1^, different magnitude responses were observed, on one occasion only dilatation and another only constriction (Fig. 3b). The average peak amplitude of dilatation was relatively low (<10% of maximum diameter; Fig. 3c) compared to what could be achieved during 15 mm KCl in the bath. To avoid the overriding complication of constriction, the same approach was utilized now using 19 mm KCl (isotonic solution) in the pipette. In these experiments, KCl stimulated local and conducted dilatation that was rapid in onset and reversal, the peak amplitude of which was approx. 20% of the maximum possible dilatation (Fig. 3d). This dilatation was fully sensitive to inhibition by the K_IR_ channel blocker Ba^2+^ (30 *μ* m; Fig. 3d). The same pipettes were then filled with a submaximal concentration of ACh (1 *μ* m) to mimic the approx. 20% local dilatation and conducted dilatation to 19 mm KCl, and these responses were not sensitive to Ba^2+^ (Fig. 3e, *n* = 3). The ability of Ba^2+^ to inhibit dilatation to bath‐applied KCl (from 4.7 mm in Mops to 19.7 mm) and ACh (10 nm to 3 *μ* m) was then assessed, with 15 mm KCl providing the greatest dilatation (Control: 60.6 ± 1.0% maximum dilatation, *n *=* *3; Fig. 3f,g). Ba^2+^ reduced the response to bath addition of KCl (+Ba^2+^: 18.8 ± 9.5% maximum dilatation) without affecting dilatation to ACh (Fig. 3f,g).

### Monophasic dilatation to luminally perfused ATP

**Figure 4:**
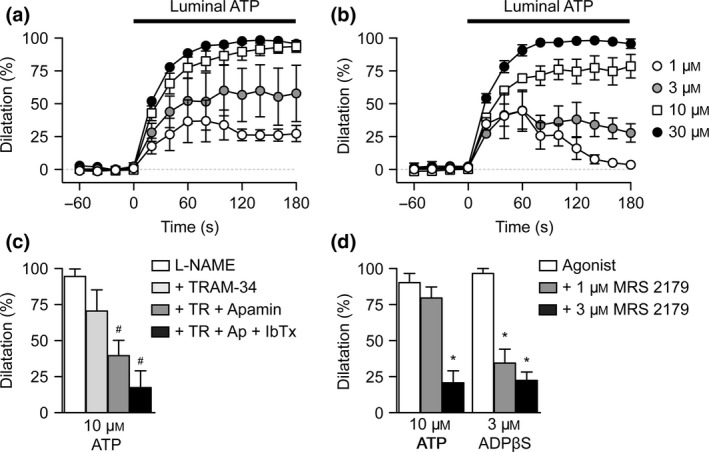
Characteristics of dilatation to luminally perfused ATP. Summary time courses of dilatation to non‐cumulative concentrations of luminally pumped ATP in the absence (a), and presence (b), of L‐NAME. (c) Summary data showing the peak response to 10 *μ* m ATP in the presence of L‐NAME (100 *μ* m,* n *=* *3) and K_C__a_ channel inhibitors TRAM‐34, apamin and IbTx (each *n *=* *3). (d) Summary data showing the peak responses to luminally perfused 10 *μ* m ATP (*n *=* *3) and 3 *μ* m ADP *β*S (*n *=* *3) under control conditions, and block by the P2Y_1_ receptor antagonist MRS 2179 (1 and 3 *μ* m, each *n *=* *3). ^#,^*P <* *0.05 vs. L‐NAME and control respectively.

To overcome the biphasic nature of the vasomotor response to ATP, ATP was continuously perfused through the lumen of arterioles. In these experiments, large‐diameter pipettes were used to reduce any increase in resistance due to pumping, and the flow rate for pumping set to 8 *μ*L min^−1^. In each experiment, the dead‐space volume of Mops buffer in the cannulating pipette took >60 s to clear, during which time there was no change in arteriolar diameter. As ATP reached the arteriole, concentration‐dependent dilatation was observed, with 10 and 30 *μ* m ATP stimulating near maximal dilatation (Fig. 4). The dilatation was only partially sensitive to inhibition of NOS with L‐NAME, when the response to 1 *μ* m ATP became more transient. The residual dilatation was blocked by the combination of TRAM‐34, apamin and iberiotoxin (IbTx), consistent with the profile of block of bath application of the endothelium‐dependent dilator ACh in rat cremaster arterioles (McSherry *et al*. 2006). A component of the dilatation to ATP was due to P2Y_1_ receptor activation, shown by block with MRS 2179 (Fig. 4d). Further evidence for P2Y_1_ receptors was shown by near 100% dilatation to the P2Y_1_ agonist ADP*β*S (3 *μ* m), an effect which was sensitive to block with 1 *μ* m MRS 2179.

### Triple‐cannulated arterioles

**Figure 5:**
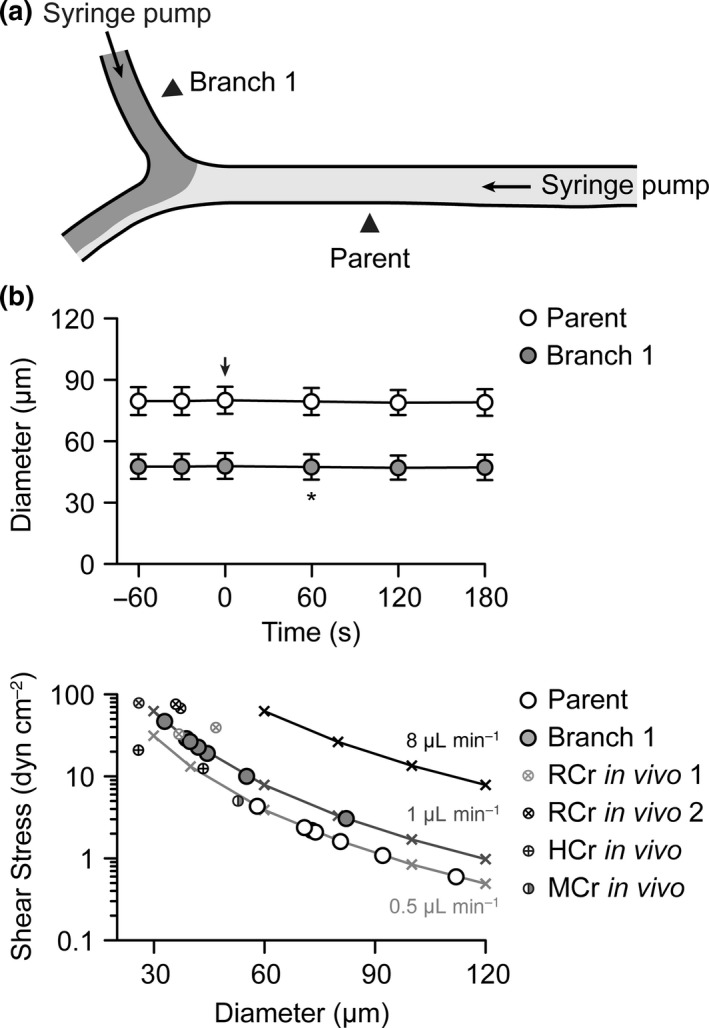
Effect of luminal flow on diameter in triple‐cannulated arterioles. (a) Schematic of triple‐cannulated arteriole set up with syringe pumps attached to the pipettes cannulating the parent and branch 1 arterioles. (b) Upper panel. Summary time course of diameter. A flow rate of 0.5 *μ*L min^−1^ was introduced into the parent arteriole at *t* = 0 s (indicated by arrow), and after 60 s, flow at 1 *μ*L min^−1^ was introduced into branch 1 (indicated by asterisk). Measurements were taken at positions indicated by arrowheads in (a). (b) Lower panel. Calculated shear stress at luminal perfusion flow rates of 0.5, 1 and 8 *μ*L min^−1^ in arterioles with diameters ranging from 30 to 120 *μ*m (indicated by crosses and lines of fit). Pump flow rates were adjusted in each experiment to avoid shear stresses above 50 dyn cm^−2^. The baseline diameters of parent and branch 1 arterioles used in the experiments with 15 mm luminal KCl (see Fig. 6) were added to these lines of fit. Published *in vivo* data from exteriorized and visualized rat (McGahren *et al*. 1997, Dora *et al*. 2000), hamster (McGahren *et al*. 1997) and mouse (Duza & Sarelius 2004) cremaster preparations are shown for comparison.

To study conducted dilatation to agonists without stimulating complicating vasoconstriction (because when receptors are also present on the vascular smooth muscle), a triple‐cannulated arteriole protocol was used as previously developed using rat and mouse mesenteric arteries (Winter & Dora 2007, Beleznai *et al*. 2011a). As the diameter of the daughter (branch) arterioles were slightly smaller than the parent arterioles, efforts were made to ensure flow *per se* had no effect on diameter. None of the flow conditions used had any effect on diameter. At these flow rates, the calculated shear stress was not greater than that observed *in vivo* in arterioles of approximately the same diameter in rat, hamster or mouse cremaster vascular bed preparations (Fig. 5).

**Figure 6:**
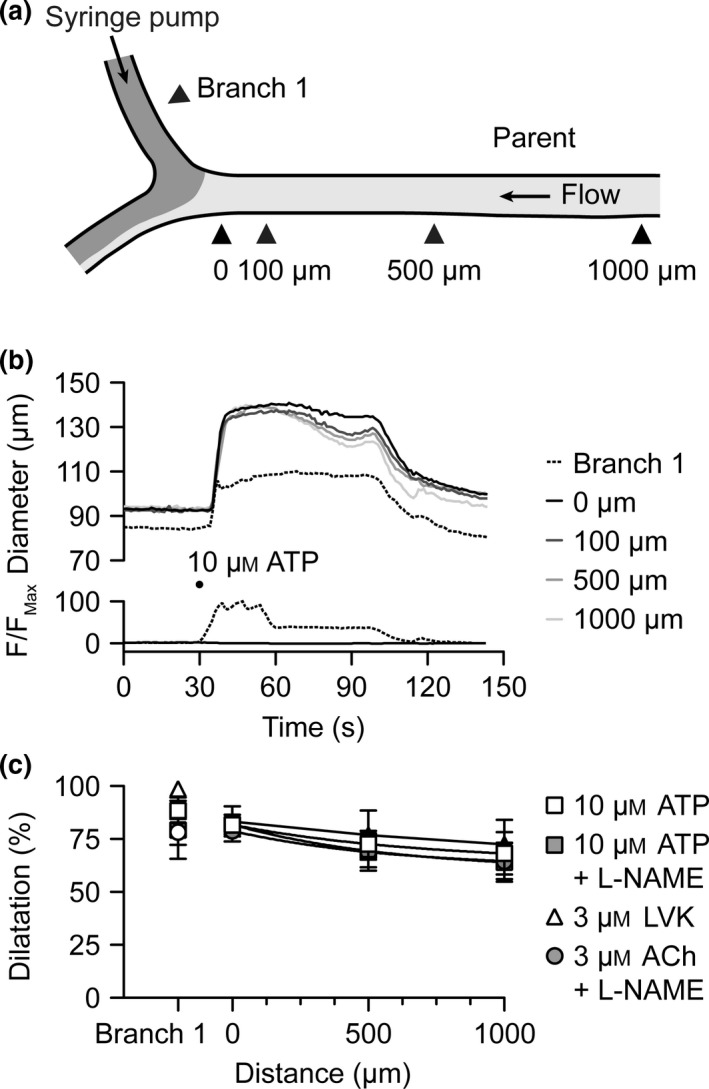
Conducted dilatation to luminal ATP. (a) Schematic of triple‐cannulated arteriole set up with a syringe pump attached to the branch 1 cannulating pipette, and flow into the parent arteriole generated by a pressure gradient (see Materials and methods). (b) Representative time course of changes in diameter and fluorescence intensity in response to luminally pumped solution containing ATP (10 *μ* m) and carboxyfluorescein. Measurements were taken in branch 1 and at sites 0–1000 *μ*m upstream [indicated by arrowheads in (a)]. (c) Summary data showing local and conducted dilatation responses to ATP and LVK under control conditions (white squares, *n *=* *5; and white triangles, *n *=* *3); and responses to ATP and ACh in the presence of the nitric oxide synthase inhibitor L‐NAME (100 *μ* m, each *n *=* *5).

Using triple‐cannulated arterioles, both local and conducted responses to agonists were obtained. Perfusion of ATP into branch 1 stimulated local and conducted dilatation with very similar magnitude to that observed to luminal perfusion of either ACh or LVK, an effect that was not influenced by the addition of L‐NAME (Fig. 6). Each agonist stimulated vasodilatation of a similar amplitude and similar decay over the 1000 *μ*m of parent arteriole upstream to the bifurcation (Fig. 6c). Compared with the other dilators, vasodilatation to LVK tended to be slower in onset and very slow to reverse, often requiring 20 min for myogenic tone to recover fully; yet the magnitude of response was not different to ACh or ATP.

### Conducted dilatation to luminally perfused KCl

**Figure 7:**
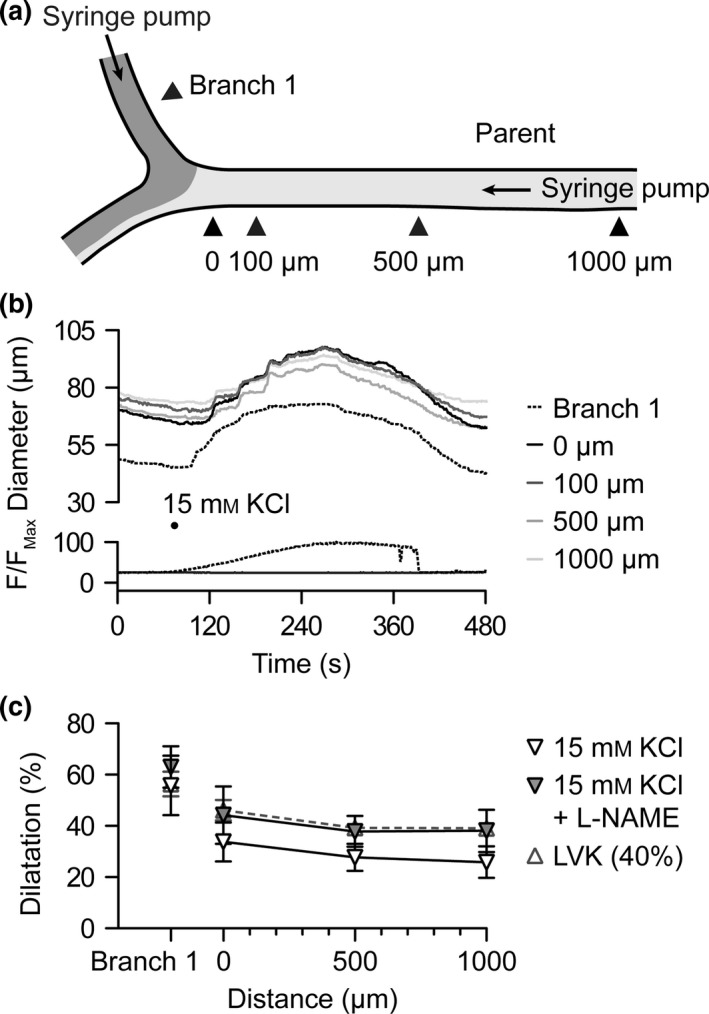
Conducted dilatation to luminal KCl. (a) Schematic of triple‐cannulated arteriole set up with syringe pumps attached to the pipettes cannulating the parent and branch 1 arterioles. Flow was first introduced into the parent arteriole at 0.5 *μ*L min^−1^ for a period of 60 s; then flow commenced into branch 1 at a rate of 1 *μ*L min^−1^ (see Fig. 5). (b) Representative time course of changes in diameter and fluorescence intensity in response to luminally pumped solution containing KCl (15 mm) and carboxyfluorescein. Measurements were taken in branch 1 and at sites 0–1000 *μ*m upstream [indicated by arrowheads in (a)]. (c) Summary data showing local and conducted dilatation responses to KCl under control conditions (white inverted triangles, *n *=* *5); in the presence of the nitric oxide synthase inhibitor L‐NAME (100 *μ* m,* n *=* *3); and for comparison, LVK during the onset of dilatation (40%). See Movie S2 for representative response to KCl.

We have previously shown that bath application of 10–15 mm KCl in rat cremaster arterioles stimulated dilatation sensitive to inhibition of Na^+^/K^+^‐ATPase with ouabain and K_IR_ channels with Ba^2+^ (McSherry *et al*. 2006), strongly suggesting hyperpolarization to KCl. In the current experiments, the flow through the parent arteriole was set constant to 0.5 *μ*L min^−1^, which was sufficient to prevent flow of KCl into the parent arteriole and ruled out any shear stress‐mediated increases in diameter. Luminal perfusion of 15 mm KCl into branch 1 at a flow rate of 1 *μ*L min^−1^ stimulated local dilatation that conducted from 0 *μ*m with little decay to the 1000‐*μ*m position in the parent arteriole (Movie S2). Note that in these experiments, use of such a low pump rate meant the onset of dilatation was slow; a consequence of mixing and washing out the buffer volume held within the cannulating pipette. Furthermore, the luminal [KCl] was likely diluted over distance along the daughter branches due to diffusion through the arteriolar wall. The resulting magnitude of dilatation in branch 1 was not as robust as to ACh, ATP or LVK, only reaching 56.8 ± 11.6% of maximal diameter (from 61 ± 7 to 87 ± 6 *μ*m, increase of 26 ± 6 *μ*m; *n *=* *5) under control conditions. This was associated with a smaller magnitude of dilatation at the 0‐*μ*m position (33.8 ± 7.6% from 89 ± 6 to 115 ± 9 *μ*m, increase of 26.1 ± 5.8 *μ*m, *n *=* *5), yet despite this, the dilatation conducted rapidly and with little decay upstream over the next 1000 *μ*m (26 ± 6%, from 93 ± 9 to 111 ± 11 *μ*m, increase of 19 ± 3 *μ*m, *n *=* *5; Fig. 7). In the same arterioles, 15 mm KCl was subsequently added to the bath, and stimulated 73.1 ± 7.1% dilatation (from 53 ± 6 to 93 ± 6 *μ*m, increase of 40 ± 3 *μ*m; *n *=* *5) in branch 1 and 61.2 ± 2.8% dilatation (from 85 ± 7 to 136 ± 9 *μ*m, increase of 51 ± 4 *μ*m; *n *=* *5) at the 500‐*μ*m position in the parent arteriole. This pattern of dilatation was not influenced by L‐NAME (*n *=* *3, Fig. 7).

### Properties of conducted dilatation in triple‐cannulated arterioles

**Figure 8:**
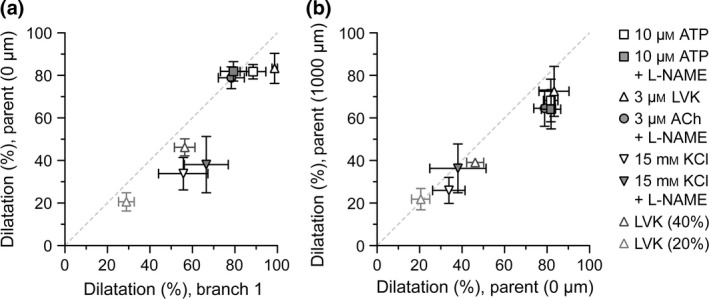
Properties of conducted dilatation in triple‐cannulated arterioles. The data presented in Figures 6 and 7 are replotted to assess the relationship between the magnitude of dilatation (% dilatation) in branch 1 and the parent arteriole (at 0 *μ*m) (a), and along the length of the parent arteriole (0 *μ*m compared to 1000 *μ*m) (b); the conducted responses. As the magnitude of response to luminal KCl was less than that to the other agonists, data during the onset of dilatation to LVK (near 40 and 20% dilatation at 0 *μ*m) were obtained for comparison. The dashed lines represent a correlation of 1.

To assess whether the drop in dilatation into the parent arteriole occurred to agents other than KCl, the LVK experiments presented in Figure 5 were re‐analysed during the onset of dilatation. Dilatation near 40% (Figs 7 and 8) and 20% (Fig. 8) of maximum at the 0‐*μ*m position on the parent arterioles was plotted for comparison. In doing so, it was established that a similar drop was also evident with LVK, another agent that directly hyperpolarizes the smooth muscle cells of arterioles. Further to this, there was no drop along the length of the arteriole to any agonist, with a near linear correlation between dilatation at the 0‐ and 1000‐*μ*m positions.

## Discussion

This study demonstrates that in isolated skeletal muscle arterioles, ATP or KCl can each stimulate both local and conducted vasodilatation. K_Ca_ channels underlie the majority of the dilatation to ATP, while K_IR_ channels are responsible for dilatation to modest increases in KCl concentration. Both mediators are generated during skeletal muscle contraction, and their ability to stimulate conducted dilatation will serve to reduce arteriolar resistance and improve blood flow into regions of ischaemia.

### Conducted vasodilatation to ACh and LVK

The ability of focal, pulsed application of ACh to stimulate rapid and reversible local and conducted dilatation is consistent with observed responses *in vivo* in rat (Dora *et al*. 2000), hamster (Kurjiaka & Segal 1995, Hoepfl *et al*. 2002) and mouse (Hungerford *et al*. 2000, Figueroa *et al*. 2003, Looft‐Wilson *et al*. 2004, Wölfle *et al*. 2011) cremaster arterioles. Once beyond the site that is stimulated by agonist, the conducted dilatation to ACh spreads with little decay between 100 and 1000 *μ*m upstream from the pipette.

The hyperpolarization underlying conducted dilatation to focal abluminal ACh is secondary to opening IK_Ca_ channels in the cannulated rat cremaster arterioles, which is consistent with studies in K_Ca_ channel knockout mouse cremaster preparations *in vivo* (Wölfle *et al*. 2009), but apparently in contrast with a role for BK_Ca_ channels in *in vivo* hamster cremaster preparations (Hoepfl *et al*. 2002). In isolated rat cremaster arterioles, we have shown a small component to ACh‐mediated dilatation that is sensitive to iberiotoxin, perhaps reflecting the release of cytochrome P_450_ metabolites (McSherry *et al*. 2006), but significantly this was not apparent in short‐pulse evoked responses used to stimulate conducted dilatation. Regardless of the K^+^ channel activated, it is clear that ACh stimulates both local and remote hyperpolarization both *in vivo* (Wölfle *et al*. 2009, 2011) and in isolated, cannulated arterioles (Emerson & Segal 2000a), relying on the endothelium as the conduction pathway enabling the spread of dilatation (Emerson & Segal 2000b).

Similarly, openers of K_ATP_ channels (pinacidil, cromakalim) have been focally applied to *in vivo* hamster (Cohen & Sarelius 2002) and mouse (Figueroa *et al*. 2003, de Wit 2010) cremaster preparations. In all preparations, the hyperpolarization underlying conducted dilatation was sensitive to glibenclamide (Cohen & Sarelius 2002, de Wit 2010). Interestingly, and in contrast to *in vivo*, here in isolated rat cremaster arterioles, the conducted dilatation to LVK did not decay more rapidly when compared to ACh (Figueroa *et al*. 2003, de Wit 2010).

### Characteristics of vasomotor responses to ATP

Application of ATP to the outside of arterioles, either as cumulative, equilibrated bath concentrations or as a bolus pulse, resulted in biphasic vasomotor responses; a transient vasoconstriction followed by vasodilatation. Although these data do not accurately reflect the true magnitude of responses, that is if they were not impeded by the opposing vasomotor responses (e.g. by damaging the endothelium), the profile is consistent with reports in other vascular beds, including penetrating rat cerebral arterioles (Dietrich *et al*. 1996, 2009, Ngai *et al*. 2007) and rat mesenteric arteries (Winter & Dora 2007).

As in rat cerebral penetrating arterioles, focal bolus application of ATP from backfilled micropipettes stimulated transient contraction and more sustained dilatation to ATP, the dilatation conducting more effectively upstream than the constriction (Dietrich *et al*. 1996, 2009, Ngai *et al*. 2007). This decay of conducted dilatation over distance is reduced following an ischaemia–reperfusion protocol (Ngai *et al*. 2007), which, although not measured directly, may reflect decay of hyperpolarization due to widespread opening of K^+^ channels (Beleznai *et al*. 2011b, Behringer & Segal 2012). In the hamster cheek pouch preparation *in vivo*, ATP stimulated a transient local constriction that was blocked by a P2X receptor antagonist, following which local application of ATP (or adenosine) stimulated local and conducted dilatation that were dependent on the endothelium (Duza & Sarelius 2003).

In the rat cremaster arterioles studied here, the transient local and conducted contraction to abluminally applied ATP presumably reflects depolarization. The more sustained dilatation to both abluminally and luminally applied ATP also rapidly and effectively conducted along the parent arteriole. The block by L‐NAME, apamin and TRAM‐34 supports actions at endothelial cell receptors, at least when luminally applied, as the targets for these inhibitors are all present within the endothelium (Bagher *et al*. 2012), and the profile of block was very similar to that observed against ACh dilatation in the same preparation (McSherry *et al*. 2006). The use of the P2Y_1_ receptor antagonist also supports endothelial cells as the primary site of stimulation, as this G‐protein‐coupled receptor is linked to the production of IP_3_ and increases in endothelial cell Ca^2+^ (Marrelli 2001, Rodríguez‐Rodríguez *et al*. 2009), and stimulation of smooth muscle P2Y_1_ receptors would cause contraction. Therefore, it is reasonable to conclude contraction follows stimulation of smooth muscle purinergic receptors, and when delivered to the lumen contraction is not stimulated at all, and dilatation occurs following stimulation of endothelial cell P2Y_1_ receptors.

### Characteristics of vasomotor responses to KCl

We and others have previously demonstrated that the dilatation observed to up to 20 mm KCl in skeletal muscle arterioles is secondary to opening K_IR_ channels and the activity of the Na^+^/K^+^‐ATPase (Burns *et al*. 2004, McSherry *et al*. 2006). The hyperpolarization and reductions in smooth muscle [Ca^2+^]_i_ were reported in isolated and cannulated hamster cremaster arterioles (Burns *et al*. 2004). When applied to the outside of arterioles, higher concentrations of KCl stimulate constriction and, depending on the method of delivery, can stimulate biphasic vasomotor responses. By adding cumulative concentrations of KCl to the bath and thereby gradually increasing the concentration, hyperpolarization and dilatation precedes depolarization and constriction (Dora *et al*. 2003), whereas focal, ejected short‐duration pulses of (1 m) KCl can stimulate rapid, transient constriction followed by dilatation (Hungerford *et al*. 2000, Budel *et al*. 2003). The latter approach was used to study conducted dilatation and indeed the dilatation conducted with variable decay over longitudinal distance (Hungerford *et al*. 2000, Budel *et al*. 2003). Here, using wider bore pipettes and continuous delivery, arterioles were exposed to concentrations more closely matching the pipette, and a lower concentration of KCl (19 mm) was able to stimulate both local dilatation and conducted dilatation, without any obscuring constriction. The dilatation was totally dependent on K_IR_ channel activation, as complete block was observed with 30 *μ* m Ba^2+^. A similar approach was utilized in porcine coronary arterioles with 10 mm KCl in the delivery pipette, in which case the local dilaation and conducted dilatation to KCl were also fully blocked by 30 *μ* m Ba^2+^ (Rivers *et al*. 2001). The advantages of this approach are multiple. In the present study, not only can a more physiologically relevant concentration of KCl be used, but the pipette position and delivery of KCl were matched before and after Ba^2+^, reducing variability between repeated stimuli. In the same arterioles, a submaximal concentration of ACh was delivered in the same manner, and although % dilatation was carefully matched, Ba^2+^ did not alter either the local or conducted dilatation to ACh. This shows that for the same magnitude of dilatation, K_IR_ channels underlie ‘local’ dilatation to KCl but do not appear to facilitate significantly conducted dilatation. Instead, other pathways must be evoked, which have yet to be defined, operating to augment the passive decay of current with longitudinal distance.

### Luminal pumping evokes monophasic local dilatation and conducted dilatation

Blood flow rate has been measured in exteriorized cremaster preparations in anaesthetized rats (McGahren *et al*. 1997, Dora *et al*. 2000), hamsters (McGahren *et al*. 1997) and mice (Duza & Sarelius 2004) and found to be below 1 *μ*L min^−1^, although others have reported values closer to 20 *μ*L min^−1^ in larger diameter arterioles (Bakker *et al*. 2003). In the present study, flow‐induced dilatation was not observed in arterioles with flow rates up to 8 *μ*L min^−1^ and shear stress up to approx. 50 dyn cm^−2^. Care was taken to avoid raising shear stress to values above 50 dyn cm^−2^, firstly as this caused irreversible damage and secondly to avoid stimulation of flow‐induced dilatation (Watanabe *et al*. 2005).

Using triple‐cannulated arterioles, it was clear that luminal perfusion‐mediated conducted dilatation to 10 *μ* m ATP was very comparable to 3 *μ* m ACh. The lack of block by L‐NAME and reliance of the residual dilatation on K_Ca_ channels suggest these K^+^ channels underlie the local and conducted dilatation. Therefore, ATP is an effective vasodilator in these skeletal muscle arterioles and is able to stimulate both local and conducted dilatation via K_Ca_ channels. Conducted dilatation to ATP has also been observed to sites over 1000 *μ*m upstream in hamster retractor muscle *in vivo* (McCullough *et al*. 1997).

Luminal perfusion of KCl also stimulated both local and conducted dilatation in triple‐cannulated arterioles. The magnitude of response in the daughter branch did not reach that observed to ACh, ATP or LVK, yet was similar in magnitude to bath application of KCl observed here and previously, approx. 60% of maximum diameter (McSherry *et al*. 2006). As the overall magnitude of dilatation was less than that observed with the other direct opener of K^+^ channels used, LVK, it appears that the magnitude of hyperpolarization was similarly less. Interestingly, as the dilatation spreads from the ‘local’ response in the daughter arteriole around the bifurcation and into the parent arteriole, there was a slight but significant drop in the magnitude of dilatation. However, this lower magnitude dilatation did not decay with distance along the arteriole (0 *μ*m through 1000 *μ*m). When the magnitude of dilatation to LVK in the daughter arteriole was matched to that of KCl, similar responses were observed in the parent arteriole (drop to, but not within). This perhaps reflects a threshold hyperpolarization necessary to cause sufficient current to generate conducted dilatation out of the daughter arteriole into the parent, so that once this threshold is reached conduction spreads with little decay during prolonged exposure to the hyperpolarizing agonist.

The presence of K_IR_ (Burns *et al*. 2004) and K_ATP_ (Jackson 2000) channels has been demonstrated in enzymatically digested single smooth muscle cells from hamster cremaster arterioles. Whether these are present in endothelial cells has not been established. In hamster retractor muscle, K_IR_ channels do appear to play a role in facilitating conducted dilatation to ACh (Jantzi *et al*. 2006). The use of a higher concentration of Ba^2+^ (100 *μ* m) was necessary to observe this contribution; 30 *μ* m Ba^2+^ was not sufficient to reduce conduction to an ACh stimulus or to fully block the dilatation to bath‐applied 15 mm KCl (Jantzi *et al*. 2006); each latter observation was consistent with the present study. However in the experiments reported here, 30 *μ* m Ba^2+^ was sufficient to abolish the dilatation to focally abluminally pumped 19 mm KCl, underlining the importance of this channel in the response to K^+^. Thus, there is an intricate interplay between the various K^+^ channels and secondary effects on K_IR_ channels and potentially also the Na^+^/K^+^‐ATPase. Whether slight changes in the resting membrane potential make differences to the contribution of K_IR_ channels to conducted responses has yet to be demonstrated; its contribution could increase at more hyperpolarized potentials (Quayle *et al*. 1996).

### Physiological relevance

**Figure 9:**
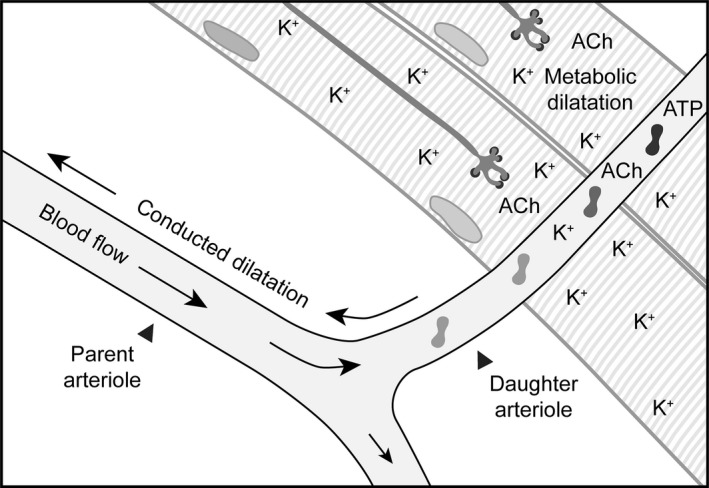
Metabolic dilatation leads to conducted dilatation to improve blood flow. Schematic depicting the release of K^+^, ACh and ATP in response to contraction of a group of skeletal muscle fibres. The extracellular [K^+^] is elevated following repolarization and hyperpolarization of the skeletal muscle fibres and motor neurones, plus the arteriolar smooth muscle and endothelial cells. The ACh released from motor neurones can diffuse to the endothelium, and ATP released from circulating cells and endothelial cells themselves can act at endothelial cell P2Y receptors. Together, these autocrine and paracrine factors can ultimately hyperpolarize the arteriolar smooth muscle cells to stimulate local ‘metabolic’ dilatation, which can spread upstream to distal sites. The conducted dilatation further reduces vascular resistance, enabling improved flow into the area of high metabolic demand. For illustrative purposes, the skeletal muscle fibres are orientated to avoid direct contact with the parent arteriole, similar to the paradigm used for studies of focal electrical stimulation of skeletal muscle bundles (Cohen & Sarelius 2002).

The physiological relevance of ATP and K^+^ as vasodilators is clear, with both acting as both autocrine and paracrine mediators (Fig. 9). When measured in the femoral vein of humans, plasma concentrations of ATP can reach micromolar levels during exercise (Rosenmeier *et al*. 2004). The potential sources for circulating ATP include red blood cells responding to low PO_2_ (Miseta *et al*. 1993, Ellsworth *et al*. 1995, Dietrich *et al*. 2000, Ellsworth 2004), platelets (Beigi *et al*. 1999) and the arterial wall itself, including the endothelial cells (Pearson & Gordon 1979, Bodin *et al*. 1991, Burnstock 1999, Yamamoto *et al*. 2003). Similarly, the levels of K^+^ in the femoral arteries and veins of humans increase by a few millimolar, depending on the level of exercise intensity (Medbo & Sejersted 1990, Vollestad *et al*. 1994, Street *et al*. 2005, Nordsborg *et al*. 2008). The concentration of each mediator within the blood of the skeletal muscle microcirculation itself may well be higher, as ATP is rapidly broken down by ectonucleotidases and K^+^ pumped back into cells via the Na^+^/K^+^‐ATPase. Measurements of [K^+^] in the interstitial space using microdialysis also report increases in [K^+^] during exercise, albeit to 9–11 mm during the most intense exercise studied (Juel *et al*. 2000, Nielsen *et al*. 2004, Street *et al*. 2005). Thus, although the reported concentrations of agents in the circulation or interstitial space in human skeletal muscle beds are slightly lower than used here, the agonists do cause dilatation at lower concentrations than used in the delivery pipettes. Therefore, the observation that either infused ATP or K^+^ stimulates vasodilatation in human skeletal muscle (K^+^ response was Ba^2+^‐sensitive) and their effects are additive (Juel *et al*. 2007) may put the current work in a more physiological context.

The ability of ACh to stimulate endothelium‐dependent local dilatation and conducted dilatation has been demonstrated in many vascular beds, yet the source of ACh and hence its relevance *in vivo* has been questioned. Within skeletal muscle, it has been shown to spillover from stimulated motor neurone endplates adjacent to arterioles to reach sufficient levels to stimulate local dilatation and conducted dilatation (Pierzga & Segal 1994, Welsh & Segal 1997). Other, potential non‐neuronal sources of ACh include circulating cells and endothelial cells (Parnavelas *et al*. 1985, Wessler & Kirkpatrick 2008), yet a clear demonstration of vasodilatation via these non‐neuronal sources within skeletal muscle beds is lacking.

The direct openers of K_ATP_ channels levcromakalim and pinacidil produce robust hyperpolarization in most vascular beds. The mechanism by which glibenclamide at least partially prevents skeletal muscle contraction‐mediated and hypoxia‐mediated vasodilatation *in vivo* (Cohen & Sarelius 2002, Ngo *et al*. 2010) has not been explained. Whether the K_ATP_ channels responsible are present within the arteriolar wall (Jackson 2000), and/or in the skeletal muscle fibres themselves (Flagg *et al*. 2010), has not been defined; but the possibility that K^+^ acts as a skeletal muscle‐derived relaxing factor, via metabolically driven K_ATP_ channels, is an area worthy of investigation.

Although the conducted dilatation to KCl was lower in amplitude than to other agonists, it would reduce vascular resistance, so the physiological relevance is still clear. Furthermore, by performing the present experiments against a membrane potential near −40 mV (Kotecha & Hill 2005), the contribution of K_IR_ channels may be limited, as K_IR_ channels are activated more effectively as the membrane becomes more hyperpolarized (Quayle *et al*. 1996, Longden & Nelson 2015), and K_IR_ channels are effective amplifiers of other K^+^ channels (Smith *et al*. 2008). Therefore, against myogenic tone alone K^+^ may not hyperpolarize smooth muscle to E_K_, nor maximally increase blood flow *in vivo*. Yet if hyperpolarization by other mechanisms were also activated (e.g. K_Ca_ and K_ATP_ channels by metabolic dilators), the combination may become very effective in improving blood flow. The cell–cell spread of hyperpolarizing current within and beyond the region of direct agonist action would then coordinate and amplify the dilatation, further reducing vascular resistance. This additive effect may not be necessary in other vascular beds where KCl has been shown to stimulate more robust dilatation, often via K_IR_ channels, particularly in the coronary and cerebral vascular beds (Knot *et al*. 1996, Rivers *et al*. 2001, McNeish *et al*. 2005, Smith *et al*. 2008).

In conclusion, we show directly for the first time that ATP or a modest increase in the concentration of K^+^ surrounding isolated cremaster arterioles can each stimulate both local dilatation and conducted dilatation. The responses, as with ACh and LVK, each follow the opening K^+^ channels present in the vascular wall. Physiologically, this suggests that in situations when these mediators are released, that is during muscle fibre contraction and/or ischaemia, each could act to facilitate an increase in blood flow by evoking a widespread drop in arteriolar resistance.

## Conflict of interest

There are no conflicts of interest.

Professor Chris J. Garland and Dr Chloe Lim provided useful critique of the manuscript. This work was supported by the British Heart Foundation (BHF) (grant numbers FS/08/033/25111, FS/13/16/30199 and IG/13/5/30431) and the Oxford BHF Centre of Research Excellence. KAD is a BHF‐funded Senior Basic Science Research Fellow. I would like to thank the Stiftelsen Nordisk Fysiologi, SNF for generous support for the AP Symposium on ‘Endothelium‐dependent hyperpolarizations 2015’.

## Supporting information


**Movie S1.** Conducted dilatation to abluminally pulsed ACh.

**Movie S2.** Conducted dilatation to luminally perfused K^+^.
